# Dr. Gooch on a Peculiar Hæmorrhage from the Uterus

**Published:** 1830-01-01

**Authors:** 


### XXII.

### On a Peculiar Hæmorrhage from the Uterus. By Dr. R. Gooch.

In Dr. Gooch's late work, there is a short chapter of a few pages on the above subject, of which the present article is a concise analysis.

When we find the uterus contracted after delivery, we do not dread hæmorrhage—nor have we, generally speaking, any cause to dread it. Yet we are often struck with the little proportion existing between the want of contraction and the degree of hæmorrhage, the uterus being sometimes found bulky without discharge of blood,—and great hæmorrhage sometimes obtaining without much size of the uterus.

"Nay further, I have witnessed a profuse hæmorrhage though the uterus had contracted in the degree which commonly indicates security; and I have ventured to do what is seldom justifiable, separate the placenta before the uterus had contracted, without more hæmorrhage than after a common labour. What is this circumstance which has so great an influence that its presence can cause a moderately contracted uterus to bleed profusely, and its absence can cause an uncontracted uterus to bleed scarcely at all?"

That the contraction of the uterus, after delivery, may occasion a sufficient closure of the blood-vessels to resist the ordinary force of the circulation, is very probable; but it is reasonable to suppose that, if the force of the circulation be extraordinarily great, the closure of the vascular orifices might be overcome, and hæmorrhage succeed. The following case is adduced to prove this.

"April 10, 1815, I delivered Mrs. S. W. of her second child; for many hours before the accession of labour she was flushed, and had a very full quick pulse. Abstinence from meat, wine, and warm drinks, a cool room, and a saline purgative, diminished, but did not remove, this state of the circulation, which continued in a considerable degree when the child was born: it was expelled very gradually, and, after the removal of the placenta, the uterus felt in the hypogastrium contracted in the ordinary degree; nevertheless, about twenty minutes afterwards there came on one of the most frightful hæmorrhages I ever witnessed; by the introduction of the hand, and the application of cold, however, it was speedily arrested.

"It was somewhat more than a year afterwards when she informed me that she was pregnant again, and coming to town to lie in. As she arrived only two or three days before she fell in labour, I did not see her till she was taken ill; but then as soon as I entered her chamber, I was struck on observing the same state of circulation that had preceded her former labour; she was sitting in her easy chair, with a red face, and a throbbing pulse. I had not been many minutes in the room before the pains became so strong it was necessary to put her on the bed, and soon afterwards the child was born; it could not be expelled more gradually; after the head was born another pain expelled the shoulders, another the body, and another the limbs. I cut the chord, placed my hand on the abdomen, and felt the uterus contracting in the usual degree, yet a few minutes afterwards the blood burst out with prodigious impetuosity. The fearful scene which followed, I need not depict; it is enough to state, that by the introduction of the hand and the application of cold, the hæmorrhage was speedily suppressed, yet it bleached her face, and for many days she could not sit up without faintness."

The inference which our author drew from these two labours was, that in this case the hæmorrhage did not depend on want of contraction of the uterus, but want of tranquillity of the circulation. He determined therefore that, in case of another pregnancy, his endeavour would be to let labour come on with a cool skin and a quiet pulse. It was not long before he had an opportunity of trying the experiment. Four months previous to her next confinement she called on our author to inform him of the event, and he advised her to take meat only thrice a week—a purgative of salts and senna thrice a week—"*a scruple of nitre three times a day:* this she began two months before she expected to be confined, and continued it up to the full time."* Four days before delivery, Dr. G. had the satisfaction to find the lady with a soft pulse and cool skin. But, unfortunately before labour commenced, the scene changed—the old heated skin and hurried circulation though somewhat mitigated, returned, and, under these circumstances, delivery took place. The surgeon put his hand on the abdomen, and said he seldom found the uterus more contracted so soon after delivery. Yet within a few minutes there came on a flooding—not so great as on former occasions, but enough to induce syncope, and detain the medical attendants in the house for several hours. In process of time this lady became pregnant again, and pursued the same plan, with the addition of a moderate venesection a fortnight before the expected accouchement, and another three or four days before that

---

* Is Dr. Gooch aware that this is an enormous dose of the nitrate of potash, continued for such a length of time? A drachm of nitre daily for two months!! We have seen much injury done to the stomach by five grain doses three times a day.—Ed.

event. She fell in labour, and Dr. G. was pleased to find the pulse soft and slow. No flooding or faintness succeeded the delivery.

"How often a disturbance of circulation plays an important part in uterine hæmorrhage it is difficult for an individual to know; but I suspect sufficiently often to deserve the especial attention of practitioners. I advise them when they meet with patients subject to hæmorrhage after delivery, to notice the state of circulation before labour, and if disturbed, to employ means for tranquillizing it before labour comes on. I advise them during labour, to use cordials cautiously, lest the placenta should separate during an excited state of circulation. I advise them after delivery, though the uterus may feel contracted, to be slow to leave their patient, if the circulation is greatly disturbed."

In hæmorrhages from the uterus, alternations of contraction and relaxation of the organ, with corresponding recurrences of bleeding, are familiar to observing practitioners. In the case of the lady above-mentioned, although the abdomen was covered with pounded ice, the hæmorrhage returned again and again, with corresponding changes from contraction to relaxation of the uterus. Finding the ice so inefficient, Dr. G. swept it off the abdomen, and poured a bason of cold water from some height upon the belly. The effect was instantaneous. The uterus contracted like a ball, and the bleeding ceased. In the second labour he took several handkerchiefs, soaked them in vinegar, and passed them, one after the other into the vagina, so as to completely fill it. This effectually prevented all external hæmorrhage. The faintness ceased, and the uterus began to harden. But these favourable symptoms did not last long. The pains ceased, the uterus grew soft and seemed to swell—the pulse became thready, and the lady grew ghastly pale. It was evident that the blood was flowing into the uterus, and that an external hæmorrhage was only converted into an internal one.

"My belief now is, that when hæmorrhage occurs after the removal of the placenta, the quickest way to stop it, is to introduce the left hand closed within the uterus, apply the right hand open to the outside of the abdomen, and then between the two to compress the part where the placenta was attached, and from which chiefly the blood is flowing. When the hand is introduced merely as a stimulant, there is an interval of time between its arrival within the uterus and the secure contraction of this organ, during which much blood is often lost. By directing the hand to the very vessels from which it issues, and compressing them as I have described, this quantity is saved. If I may judge by my feeling, the blood stops, in a great degree, even before the uterus contracts: the hand acts first as a tourniquet, then as a stimulant. It is true we cannot tell with certainty where the placenta was attached, and consequently where the pressure should be applied; but as it is generally attached to or near the fundus, if the pressure be directed there, it will generally be right."

But to return to the patient, whom we left nearly in articulo mortis. Dr. G. drew out the handkerchiefs and applied his hands in the manner above described, with the most immediate and happy effect. The bleeding stopped—the patient revived—and the uterus contracted. She looked blanched and pale for several days afterwards.

---

